# Research informatics and the COVID-19 pandemic: Challenges, innovations, lessons learned, and recommendations

**DOI:** 10.1017/cts.2021.26

**Published:** 2021-03-16

**Authors:** Richard J. Bookman, James J. Cimino, Christopher A. Harle, Rhonda G. Kost, Sean Mooney, Emily Pfaff, Svetlana Rojevsky, Jonathan N. Tobin, Adam Wilcox, Nick F. Tsinoremas

**Affiliations:** 1 Department of Molecular and Cell Pharmacology, Clinical and Translational Science Institute, University of Miami, Miami, FL, USA; 2 Informatics Institute, Center for Clinical and Translational Science, University of Alabama at Birmingham, Birmingham, AL, USA; 3 Department of Health Outcomes and Biomedical Informatics, Clinical and Translational Science Institute, University of Florida, Gainesville, FL, USA; 4 Center for Clinical and Translational Science, the Rockefeller University, New York, NY, USA; 5 Institute for Translational Health Sciences, University of Washington, Seattle, WA, USA; 6 Department of Medicine, North Carolina Translational and Clinical Sciences Institute, University of North Carolina at Chapel Hill, Chapel Hill, NC, USA; 7 Clinical and Translational Institute, Tufts Medical Center, Boston, USA; 8 Clinical Directors Network (CDN), the Rockefeller University Center for Clinical and Translational Science, New York, NY, USA; 9 Department of Biomedical Informatics and Medical Education, Institute for Translational Health Sciences, University of Washington, Seattle, WA, USA; 10 Department of Biochemistry and Molecular Biology, Clinical and Translational Science Institute, University of Miami, Miami, FL, USA

**Keywords:** CTSA, COVID-19, informatics, N3C, research

## Abstract

The recipients of NIH’s Clinical and Translational Science Awards (CTSA) have worked for over a decade to build informatics infrastructure in support of clinical and translational research. This infrastructure has proved invaluable for supporting responses to the current COVID-19 pandemic through direct patient care, clinical decision support, training researchers and practitioners, as well as public health surveillance and clinical research to levels that could not have been accomplished without the years of ground-laying work by the CTSAs. In this paper, we provide a perspective on our COVID-19 work and present relevant results of a survey of CTSA sites to broaden our understanding of the key features of their informatics programs, the informatics-related challenges they have experienced under COVID-19, and some of the innovations and solutions they developed in response to the pandemic. Responses demonstrated increased reliance by healthcare providers and researchers on access to electronic health record (EHR) data, both for local needs and for sharing with other institutions and national consortia. The initial work of the CTSAs on data capture, standards, interchange, and sharing policies all contributed to solutions, best illustrated by the creation, in record time, of a national clinical data repository in the National COVID-19 Cohort Collaborative (N3C). The survey data support seven recommendations for areas of informatics and public health investment and further study to support clinical and translational research in the post-COVID-19 era.

## Introduction

Over more than a decade, NIH’s National Center for Advancing Translational Science (NCATS) has funded Clinical and Translational Science Awards (CTSA) at more than 60 institutions in the USA. These institutions have worked together to build informatics infrastructure in support of local and national research initiatives. A key infrastructure element at each site has been a clinical data repository, drawn from the site’s electronic health records (EHRs). These repositories support research activities such as cohort estimation, natural history studies, and interinstitutional sharing of retrospective data, as well as provide a foundation for ongoing evaluation and feedback to build a learning health system.

These efforts have taken place during a time of relative quiet in terms of public health emergencies. The start of the HIV/AIDS epidemic predated the CTSA era by decades and intervening scares such as MERS, SARS, and H1N1 were of modest scale. The emergence of SARS-CoV-2 and COVID-19 in late 2019 presented extraordinary and sustained challenges in epidemiologic monitoring, new disease discovery, basic and clinical research, support for diagnosis and care of individual patients, and population prevention activities. The CTSA clinical data repositories have been at the right place and the right time to support these efforts.

The initial wave of operational and clinical innovation to support demand for both COVID-19 and non-COVID-19 care was immediately followed by a surge in demand for informatics (IT) resources to support clinical operations, public health surveillance, and to support COVID-19-related research. To meet the demand for critically needed research about an emerging infectious disease, research informatics teams had to adapt quickly and with a high degree of flexibility and innovation [1]. Support for this type of research required the generation of reports, dashboards, and datasets using common data models (CDMs) for data harmonization and normalization in near real time, putting to the test the value and utility of tens of billions of dollars that the US healthcare system has invested in EHRs [2].

In this communication, we pause mid-pandemic and offer our combined perspectives on the support that research informatics cores provided to their organizations in response to the demands of the current public health emergency. We represent seven CTSAs from across the country – some with early hotspot experience, others from locations that are only recently experiencing the third wave. To broaden the perspective, we developed a survey and collected responses from 95% (60/63) of CTSAs to provide a fuller picture of the pandemic response by the research informatics teams. We identified key areas of challenge, of innovation, and of learning at these CTSA institutions. Those results are presented in the “Survey Results” section.

We first want to highlight key features of the CTSA consortium’s informatics programs that were already in place by late 2019. We then describe the range of informatics-related challenges experienced at CTSA hubs during the first 8 months of the COVID-19 pandemic and some of the solutions and innovations sparked by these new demands. All this culminated in the rapid deployment of the National COVID-19 Cohort Collaborative (N3C), which we will briefly describe. We then present the results of our survey of CTSA institutions’ pre- and post-COVID-19 informatics resources and challenges. The pandemic has afforded insights into areas of informatics where additional innovations are needed. We, therefore, distill themes and discuss some lessons learned that we hope can help us prepare for later stages of this pandemic and future ones. Finally, we offer a series of recommendations that may help CTSAs continue to accelerate translational research while responding to the demands of the pandemic as novel diagnostic and screening tests, treatments, and multiple vaccines become available.

## The Pre-Pandemic Research Informatics Environment at CTSAs

The CTSA consortium has been working together for years to build an informatics infrastructure to support collaborative clinical and translational research. By way of the survey, we reviewed the “CTS environment” in the academic medical centers before the pandemic and the extent to which they were prepared for the challenges as the pandemic unfolded. In terms of informatics, we highlight seven areas:

- Governance: We observed varying levels of maturity of the formal processes for (i) allocation of scarce resources and (ii) data sharing. Generally, these processes were not designed to and did not work rapidly and in times of peak demand.
- Tools: The CTSAs have built shared informatics tools with varying degrees of adoption. The positive example of REDCap [3] shows the value of widespread adoption of a general purpose, versatile database design, and management tool [4].
- CDMs: *CDMs are needed to centralize or federate multiple sources of data. For EHR data, many have emerged over the last 15 years* – and five now have a prominent place [links embedded] – Observational Medical Outcomes Partnership (OMOP), FDA Sentinel, Patient-Centered Outcomes Research Network (PCORNet), Informatics for Integrating Biology and the Bedside and Accrual to Clinical Trials network (i2b2/ACT) [5], and TriNetX [6]. While CDMs enable multisite, data-driven research, the implementation and maintenance of even one CDM consume human and other resources. Our survey results show that CTSAs are supporting an average of three CDMs.
- Data terminology standards: *Heavy reliance on standards is common from ICD-10 to LOINC, SNOMED, HL7, etc.* [7,8] However, any standard terminology is needed for a new disease, whether for CDMs or phenotyping takes time to be formalized, disseminated, adopted, and implemented.
- Training: *Informatics training has been a focus for CTSAs from the start. To the extent that a CTSA has reached a certain level of informatics self-reliance among its users, the ability of the informatics core team to support the surge in requests is made easier, thereby contributing significantly to institutional agility.*
- Participant protections: All CTSAs are migrating to the use of a central IRB for multisite studies. Local IRBs may lack experience in reviewing and approving remote consenting, remote research visits, and other applications of telehealth and mHealth technologies whose use in the pandemic has grown.
- Research use of the EHR: *There is significant variation across CTSAs with respect to EHR integration to serve research needs. Experience with cohort identification, composing and validating computable phenotypes, and populating patient registries is valuable* [9] User-friendly tools to evaluate cohorts unburden informatics teams and encourage investigator-level familiarity with and exploration of EHR data.

## Informatics During COVID-19: Challenges and Solutions

Despite this solid background of accomplishments, the pandemic precipitously and profoundly affected the conduct of clinical research across the CTSAs and the world. By requiring physical distance between clinicians and patients, investigators, and research participants, research was forced overnight into the virtual space, urgently requiring innovation and expanded support from informatics teams within each institution and across the CTSA consortium. One major theme that we detect running across many of these actions is the speed with which, under pandemic pressure, decisions were made. At our institutions, risk tolerance, particularly with respect to telemedicine, was recalibrated. Decision-making timelines were shortened.

Public health directives to minimize in-person interactions to reduce the risk of COVID-19 transmission created a surge in demand for innovations to support remote interactions with patients. With rapid coverage changes made by CMS and insurance providers that allowed for reimbursement of telehealth visits, many concerns about reimbursement rates were minimized. The need for social distancing and for protection of patient and healthcare worker safety required the rapid uptake of telehealth services at a new scale for both urgent and routine care visits as well as for research visits. This was also essential to support ongoing, non-COVID-19-related studies. Remote clinical and research visits, and the electronic conduct and capture of informed consent (eConsent) moved from being a futuristic option weighted by privacy concerns to a state of urgent and high demand followed by immediate adoption and scale-up. Importantly, many pre-COVID-19 barriers to the implementation of these activities rapidly fell away as institutional leadership, administrators, regulators, and researchers fell into unprecedented alignment of purpose. Technical projects that previously were predicted to require many months or longer were completed in weeks. Operational challenges of access to technology, personnel, bandwidth, and training were matched by informatics challenges to integrate platforms (Clinical Trials Management Systems (CTMSs), EHR, video teleconferencing), create biorepositories, conduct cohort identification, etc.

Universally, we observed that investigators requested platforms and technical solutions for not only virtual research visits, but remote conduct of informed consent and for electronic capture of the interaction. There are limited available platforms for eConsent, some of which are expensive to license or complex to configure. We observed that the majority of CTSA organizations use DocuSign (https://www.docusign.com/), REDCap Cloud, (https://redcapcloud.com/), and REDCap for nonprofit organizations (https://projectredcap.org/). The FDA released its own eConsent platform, (https://www.fda.gov/drugs/science-and-research-drugs/covid-mystudies-application-app) which is limited to FDA-regulated COVID-19 studies, generating both inconvenience and complexity for informatics teams supporting the operation and integration of multiple platforms for eConsenting. None of the available eConsent platforms was interoperable with common CTMSs or EHR platforms. As there was limited use of eConsent pre-pandemic, there is a limited evidence base regarding the potential impact of the shift to remote consent platforms on human research protections, equitable access to research, or the potential impact on the demographics of cohort identification, enrollment, and representative research [10]. As eConsent is conducted through individual secure login accounts, there may be insufficient tracking of virtual versus in-person consent and data capture to support robust comparisons. Vanderbilt’s *eConsent* (*REDCap eConsent framework*) is the dominant source for a low-cost eConsent platform within the consortium [11]. Challenges and innovations are summarized in Table 1.

**Table 1.** Challenges, response, and innovations in informatics during COVID-19

Informatics resource	Pre-pandemic challenges	Intersection with research activities	Impact of pandemic	Lowering of barriers	Considerations
Telehealth (care)	– Reimbursement problematic	– Means of capture of healthcare data used in research	– Surge in demand for virtual visits	CMS waivers reimbursement	Telehealth proved acceptable, or even preferable for many patients and providers. It will persist after COVID-19.
	– Limited demand	– Integration of wearables and other remote data capture (vital signs, EKG, glucose, etc.)	– Need to rapidly develop policy, resource allocation, data sharing, and secure means of patient–provider communications	https://www.cms.gov/files/document/covid-19-physicians-and-practitioners.pdf	Need for research to determine in which settings delivery of care by telehealth is equivalent, inferior, or superior compared to in-person care.
	– Assumption that in person is preferred	– *How does this intersect with the shifting computable phenotypes for COVID-19 research?*	– Broad adoption and acceptance	AMC/University officials aligned with priority to expand access to remote care	
	– Requirement to collect in-person vital signs at each visit		– Operational priorities at the expense of strategic design		
Telehealth (research virtual visits)	– Limited demand	– Applicable to at least part of all research	– Surge in demand	IRB/university/research teams all aligned to accelerate policy and approval	Likely sustainable; not subject to any CMS funding reversal.
	– Assumed participant and researcher preference for in-person visits	– Conduct of research virtual visits; requires integration with data capture platform, CTMS?			
	– IRB approval as per protocol exception				
eConsent	– Reluctance from administrators, legal, IRB, research teams, informatics to develop infrastructure for eConsent	Distinct privacy and regulatory frameworks for non-FDA and FDA-regulated research	Surge in demand	IRB/university/IRB/research teams all aligned	Likely sustainable, more and better options will become available to researchers as the niche expands
	– Assumption that in person is better	– Need for integration with other systems (e.g., EHR) to extract research data	– Need to develop secure and compliant infrastructure, policy SOP, oversight, training	Resources for informatics to support/build eConsent and virtual visit frameworks Training and support barriers persist as eConsnet framework is complex and demanding, even as it is improving	Research need: What are the gaps created or filled by eConsent compared to prior practice? Which types of studies or participants are best served by eConsent?
	– Reluctance to change standard operating procedures to allow for electronic consent and still meet regulatory requirements		– Integration with existing systems; multisite platforms		
	Absence of the reasonably priced and compliant option to administer eConsent				

The quality, depth, and breadth of data collected by healthcare organizations have provided increasing opportunities to advance knowledge and population health, especially during the COVID-19 pandemic. To be able to make sense of the information contained in EHRs and data warehouses, the scale is an absolute necessity. That scale makes it possible to ask and answer important questions, provide evidence for policy changes in the standard of care, care delivery, coverage structure, and emerging population health trends. Further, the required scale is, in the USA, rarely achieved by any single health system. Thus, scale requires that data must be aggregated, and this requires consistent use of standard definitions and terminology.

## The National COVID-19 Cohort Collaborative (N3C)

Prior to the COVID-19 pandemic, the proliferation of CDMs for EHR data across the CTSA consortium was already changing the landscape of multisite, data-driven clinical research. Benefits of CDMs include the ability to share code for phenotyping, analysis, and predictive analytics; consistently defined, EHR vendor- and institution-agnostic clinical variables; and the availability of well-documented data quality assurance scripts.

**Fig. 1.**
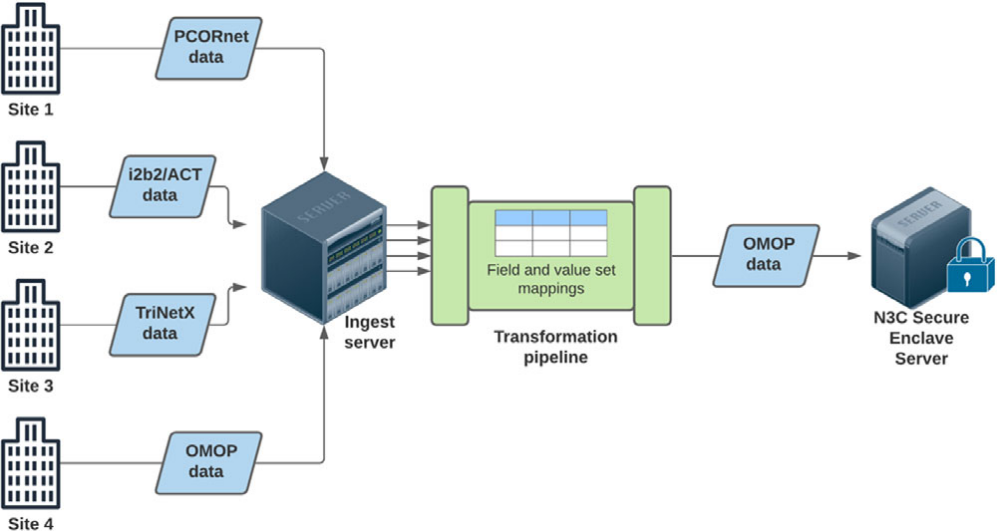
N3C sites can submit EHR data for their COVID-19 population in any one of the four data models. Once transmitted to NCATS, a transformation pipeline maps fields and value sets from the source data models to the OMOP data model. In the near future, privacy-preserving hashing methods will allow for some deduplication of patients as part of the pipeline. Harmonized data in the OMOP model are made available to researchers in a secure analytics enclave. N3C, National COVID-19 Cohort Collaborative; OMOP, Observational Medical Outcomes Partnership; i2b2, Informatics for Integrating Biology and the Bedside; ACT, Accrual to Clinical Trials network; TriNext, company named TriNext.

Despite the advantages of CDM infrastructure, the five major CDMs (OHDSI/OMOP, i2b2/ACT, Sentinel, PCORnet, TriNetX) do not readily interoperate with each other. If institutions that wish to share data do not support the same CDM – perhaps one uses i2b2/ACT, while another uses OMOP – there are very few options available to bridge that semantic and structural gap. Moreover, due to the fact that mapping between CDMs is resource- and personnel-intensive, there has historically been little institutional will to launch full-scale efforts to do so. Thus, when NCATS devised a plan for a centralized, nationwide EHR data repository for COVID-19 research, it was necessary to overcome this long-standing barrier. When designing its repository, N3C ultimately chose to harmonize four CDMs to OMOP as shown in Fig. 1.

N3C enables broad access to harmonized EHR data to support community-driven, reproducible, and transparent analyses of COVID-19 data (Fig. 1). Key to N3C’s architecture and success is the capability to map and transform between CDMs. Clear prioritization from NIH/NCATS and the weight of the pandemic both contributed to institutions’ willingness to expend resources on mapping data models, working out governance and regulatory challenges, and extracting data for contribution. At the time of this writing, the N3C repository contains EHR data from 41 sites and over 3 million patients, representing large-scale harmonization of 4 CDMs into a single dataset. After regulatory and governance approval, investigators can access and analyze these data in a secure enclave for COVID-19-related research questions. As of this writing, 164 institutions have signed the Data Use Agreement (DUA) that allows their researchers to access the enclave for approved projects [12].

## CTSA Informatics Survey

### Methods

As one of the several writing teams for this special issue, we wanted to broaden our own experiences by soliciting the views of others. We developed survey questions related to operational disruptions of research and changes due to COVID-19. For the purposes of this paper, questions focused on research infrastructure, disruptions, and changes related to data, information technology, and other informatics processes and tools. Specifically, we asked objective questions related to the timing of research operations disruptions, research data warehouse and CDM infrastructure, research data request processes, COVID-19-specific research data infrastructure and processes, and informatics-related changes and innovations that stemmed from the pandemic. Survey questions were reviewed by the authors, representing six institutions, and modified such that they would be relevant and answerable at all institutions The survey was disseminated to all CTSA hubs electronically via REDCap between (September 22, 2020) and (October 7, 2020). Survey questions are available in the Supplementary Materials.

### Results

In total, 60 CTSA hubs responded to the survey, although some did not complete all questions. When asked to indicate the period in which their hub began to experience COVID-19-related research operations disruptions, 82% (*n* = 49 out of 60) reported research operations began to be interrupted by COVID-19 in the January–March timeframe. Ten hubs (17%) reported disruptions beginning in the April–June timeframe, and one hub responded “not applicable” to this question.

In terms of pre-COVID-19 research data infrastructure, 95% of hubs that responded to the question (*n* = 56 out of 59) reported having a dedicated research data warehouse. Of 56 hubs that reported using 1 or more CDMs, 50 (89%) reported using i2b2, 40 (71%) reported using OMOP, 32 (57%) reported using PCORNet, 32 (57%) reported using TriNetX, and 13 (23%) reported an “other” CDM. On average, an institution is supporting 3 CDMs, and 38/56 reported using 3 or more. When asked to report their pre-pandemic research data warehouse refresh rates, hubs responded monthly (*n* = 23 out of 56, 41%), weekly (*n* = 8, 14%), daily (*n* = 16, 29%), or “other” (*n* = 9, 16%). Thirty out of 56 hubs reported increasing their rates of data warehouse refreshes during the pandemic.

Fifty-four of 59 hubs that responded to the question (92%) reported that they can now support rapid cohort identification of COVID-19 patients for research, and 51 (86%) of hubs reported they developed a “COVID-19 Data Mart/Registry and/or a dashboard.” Reported uses of these data marts, registries, or dashboards included predictive modeling (*n* = 42 out of 50, 84%) clinical trials (*n* = 39, 78%), operations (*n* = 43, 86%), and “other” (*n* = 20, 40%).

Fifty-one of 58 hubs that responded to the question (88%) reported increased utilization of informatics resources due to COVID-19, such as REDCap, data warehouses, and CTMSs. Six hubs (10%) reported no change in utilization, and one hub (2%) reported a decrease in utilization.

None of the survey questions asked explicitly about the implementation of infrastructure to support remote consent or eConsent infrastructure. However, multiple questions produced responses that were relevant. In responding to a survey question about how the increase in biorepository demands affected the institution, 42 of 60 (70%) respondents mentioned the institution or expansion of eConsent capacity as an important effect. In responding to questions about virtual visits (some of which may have necessitated eConsent), 19 out of 52 (35%) reported preexisting infrastructure for virtual visits, and 25 out of 52 (47%) reported instituting new processes (infrastructure) that they planned to keep after pandemic demands recede. Ten out of 64 reported obtaining consent as the biggest challenge to virtual visits; while 26 of 64 mentioned consent in qualitative responses to a request to list leading innovations implemented as a result of the pandemic.

Thirty-eight of 58 hubs that responded to the question (66%) reported having a committee to manage the process for prioritizing access to EHR data. Of those 38 hubs, 9 (24%) reported that the committee was new (or expanded) during the pandemic.

## Necessary Innovation, Lessons Learned, and Recommendations for Future Study

The USA and our academic medical centers were inadequately prepared for the COVID-19 pandemic, despite >$35B spent on electronic medical records, decades of warnings of the threat of pandemics, and significant public investments in emergency preparedness for chemical, biological, radiological, and cyberattacks. In numerous cities and regions, the healthcare delivery system was or is severely strained. The current crisis is far from over: as this is being written, we are in the third wave in COVID-19 cases in the USA and elsewhere. In December, the first two highly effective vaccines received FDA Emergency Use Authorization and their administration began to those at highest risk. By mid-February, more than 40 million doses have been administered in the USA. Adequate suppression of the pandemic to return to some normalcy, given the widespread use of effective vaccination coupled with public health measures, is thought to be still half a year or more away. The pandemic has been a stress test to the system, and it’s useful to consider where strengths and weaknesses were identified so that our institutions are better prepared to respond to the next challenges of the current pandemic and to manage better when the next one strikes. In our increasingly digital world, the centrality of data to health has never been more clear or important. Our data performance has shown numerous gaps in data, data quality, and data flow.

Pre-pandemic, the informatics organizations of the CTSA network were focused on conducting and supporting diverse areas of biomedical research. As shown above, most groups developed processes and tools to handle hundreds of research projects. During this time, almost everyone had to support multiple standards for health and biomedical data and had to develop distinct ways of sharing those data with each of the respective networks to which they belonged. Furthermore, many of the teams were participating in and/or supporting projects with tools and analytics that were designed for interventional clinical and community outreach projects. In March2020, nearly all research studies halted and nearly all research and operations shifted to support COVID-19 patients and COVID-19-related projects in real time. Due to the nature of the pandemic, the speed of change is unprecedented at every level.

We can start illustrating this in the area of governance. Typically, DUAs and Memoranda of Understanding (MOUs) require months of back and forth refinement. Now, driven by clinical needs, these have been arranged in a matter of weeks. What exactly was the value of all those rounds of back and forth? Confronted by a life-threatening pandemic, some of those concerns gladly receded into the background. Datasets are now being shared with multiple stakeholders within and outside our institutions while still complying with regulations and protecting patient privacy. How can we learn from this and extend the agility of data sharing under COVID-19 to be a routine feature of our post-pandemic life? Privacy protections are and will remain important, but we recommend including some extended protections to data custodians under-declared public health emergencies.

Rapid data-sharing agreements are clearly a feature of operating in this pandemic. But, such flexibility only increased the speed with which organizations needed to consider issues of both technical and semantic interoperability, as well as data quality. New datasets from a number of different sources, such as the state-level immunization registries, Health Information Exchanges (HIEs), claims databases such as CMS (Medicare, Medicaid) and all payers’ databases need to be linked, evaluated, and studied. There has been a significant challenge with the collection of the data, and modeling and analysis of the data attributed to different individuals collected under difficult situations. The pressure of the pandemic does not allow much in-depth communication to understand the overall objectives.

COVID-19 testing databases and COVID-19 immunization registries capture numerators while EHRs integrated across multiple healthcare systems, e.g., through HIEs, capture one key denominator that reflects persons in care. The true population denominator, necessary for accurate population-based estimates, might be census-based, or some combination of social security, passport/visa, and immigration registration-based data sources, or an eventually, a national patient ID.

Since many patients have or will receive COVID-19 testing data and COVID-19 vaccination from providers that are not their usual sources of care, ensuring that the test and immunization data are transferred into consolidated medical records is important for calculating overall population and population subgroup rates. This requires the de-duplication and integration of these multiple data streams at the individual patient level, as well as careful capture not only of the event data, but also the metadata (type of test, vaccine manufacturer, lot number, etc.). Data integration efforts that combine such numerator and denominator information are critical for several purposes, including:

1. Public health – establishing vaccine coverage rates with the granularity (e.g., demographic and geographic data) to examine subgroup reach and to target underserved/underrepresented groups (especially those individuals and groups who may be excluded systematically or who avoid healthcare settings).
2. Pharmacovigilance – to capture vaccine adverse events, some potentially rare, subtle, and unrecognized and to have demographic and clinical information to examine the heterogeneity of treatment effects (HTE) in demographic and clinical subgroups.
3. Clinical – to evaluate up-to-date status for clinical preventive services so clinicians at the point of care can help patients become and stay up to date for indicated preventive services (e.g., get tested, receive second dose, and probable booster doses, etc.) and to capture longitudinal information on infection incidence, severity, and outcome, especially to characterize, phenotype, and treat long-haul COVID-19.

New natural history and clinical outcomes studies had to be set up in record time, while data and data standards were often changing as IRB protocols were being written. An example is laboratory data. New COVID-19 tests are being approved (or authorized under a EUA) frequently, producing new definitions, new LOINC codes, SNOMED terms, values, etc. Studies to evaluate those tests, in terms of sensitivity, specificity, etc., are still in the early stages. The US Department of Health and Human Services offered a solution. SHIELD (https://aspe.hhs.gov/shield-standardization-lab-data-enhance-patient-centered-outcomes-research-and-value-based-care) offers a standard for reporting lab data but many organizations are still not aware of this standard, let alone implementing it. The N3C effort, by tackling the CDM interoperability challenge as described above, has helped substantially. Now, there is better coordination and many are working toward common goals. This is an effort we expect to continue through the pandemic and beyond since it is an essential part of health informatics preparedness.

Faced with an unknown new disease with no preexisting coding, we relied on standard-setting bodies (for ICD10, LOINC, SNOMED, etc.) to rapidly provide new codes and terms. However, the variables that investigators want to work with are often not the same variables that are coded and standardized – e.g., visit types, ventilator settings, ICU admissions and length of stay, oxygen saturation, and symptoms. This speaks to the need for more agile standardization for certain use cases – a “good enough” approach that may lack in the robustness of HL7 or SNOMED, but does not rely on lengthy deliberation and approval processes. Some of the CDM communities took steps in this direction in response to COVID-19; PCORnet quickly developed standard ways of representing certain COVID-19-specific variables, such as ventilator and ICU flags, and the ACT ontology devised its own system of flagging positive COVID-19 lab tests (see https://github.com/shyamvis/ACT-COVID-Ontology/blob/master/ontology/README.md).

Informatics groups are in the middle of all of these rapid changes. Multiple demands and resource constraints are very real. Most groups had to innovate and adapt fast. As indicated in our survey, many groups developed dashboards, COVID-19 data marts, standardized extracts, and deployed eConsent digital tools for clinical trials. They also developed new data models and participated in research to evaluate the process and clinical outcomes of telehealth encounters as compared to face-to-face visits. Such dramatic changes to the delivery of health care are only now being rigorously evaluated. The effectiveness of these changes and innovations is yet to be ascertained, and the research has already started. Informatics groups have to be ready to respond to this challenge, including developing new, agreed-upon metrics for the process of care that can be captured passively or with minimal interference with care.

As mentioned earlier, investigators needed to conduct the informed consent process remotely and to capture the interaction electronically. A majority of institutions expanded or developed new eConsent infrastructure and most will be keeping it. Management of eConsent for FDA-regulated studies frequently requires the support of a separate platform adding complexity for both users and informatics teams. Due to the lack of interoperability with common CTMS or EHR platforms, continued innovation and harmonization will be needed in this area. We also want to emphasize that the potential positive and negative impacts of remote consent procedures on human subject protections, engagement of hard-to-reach populations, and the role of the digital divide inequitable and representative research will be important topics for near-term research inquiry and debate.

Finally, we are still in the middle of the pandemic and many additional challenges are yet to come. Some are just around the corner and will require massive informatics efforts that go beyond our individual institutions. The evolving N3C resource should prove to be a valuable asset to a diverse set of investigators. But, the more “typical” research project might now involve state and national immunization registries, real-time immunization information, record linkage, data fusion, data curation, and machine-to-machine understanding of adverse effects. We, therefore, anticipate new challenges in the next phase of battling the pandemic. This leads us to make some recommendations.

1. There is a fundamental and perhaps somewhat underappreciated difference between clinical informatics and public health informatics. It seems fair to say that the CTSAs, by and large, have been evolving in the ecosystem of clinical and translational research, and not public health research. As a result, many – perhaps most – were and are unprepared to quickly integrate with preexisting public health data infrastructure and respond to public health research needs. Further, as we have explored our local or state public health data infrastructure, we are concerned that significant investment is needed to raise the level of maturity and agility of many public health information systems. These gaps need immediate attention as many COVID-19 vaccines will become available over the next year or two. Collaborating with existing organizations, many of them at the state level will be both mutually beneficial and necessary. Focusing on the data sharing and DUAs with state immunization registries and clinical laboratory testing data systems, perhaps at the national level with the CDC to avoid 64-fold repetition, can help to implement a safe, equitable, and effective mass vaccination campaign.
2. We see real value in examining “data preparedness” and “data logistics” in declaring public health emergencies [13]. Imagine if there were an equivalent to the Federal Emergency Management Agency (FEMA) for health data that could pre-position assets at the earliest phase of a pandemic.
3. We urge the NIH to support research to refine culturally appropriate best practices and privacy guidelines as healthcare research moves to a more virtual/distanced world. From eConsent to home device monitoring, both care and research will be more of a noncontact affair in the future. This shift is worthy of study in and of itself so that we have the evidence to refine our distanced approaches to both care and research in a patient-centric way.
4. This rethinking of “place” also has interesting implications for talent development and staffing. As informatics skills, particularly in areas like artificial intelligence (AI) and machine learning, are in such high demand, academic institutions feel at a disadvantage. HR policies should be reevaluated to seek ways to broaden applicant pools by supporting WfA – Work from Anywhere – telecommuting and remote work policies, while maintaining the deep collaboration required by academic work.
5. The COVID-19 pandemic has put all the CTSAs on high alert status, working as fast as possible to be of service. It is fair to say that “pandemic preparedness” is not a standard part of CTSA evaluation. We encourage NCATS to begin a series of discussions with CTSA leadership on this topic. A workshop in 2022 to layout the plans for the next pandemic would help the CTSAs consolidate and share lessons from this one and highlight to NIH leadership potential areas for further infrastructure investment. It will be important to engage other DHHS entities, such as CDC, FDA, ONC, CMS, and HRSA in these discussions. “Dual-use” informatics can help both clinical research and serve public health needs.
6. In the spirit of never wasting a good crisis, it is worth reviewing those processes that previously required months and hours of meetings. What is the real value of those steps? Let’s not slip back?
7. Regarding disparities and equity, That COVID-19 does not impact all communities equally in the USA was a fairly early observation and soon became a substantiated finding. It’s time to ask ourselves – “How did we do?” – and be prepared to spend the time to answer it rigorously and then commit resources, including in informatics, to do better for all phases of pandemic response.

## Supplementary material

For supplementary material accompanying this paper visit http://dx.doi.org/10.1017/cts.2021.26. click here to view supplementary material
