# Supplementary material for: Research informatics and the COVID-19 pandemic: Challenges, innovations, lessons learned, and recommendations
Source: J Clin Transl Sci. 2021 Mar 16;5(1):e110. doi: 10.1017/cts.2021.26 (PMC8209435; doi:10.1017/cts.2021.26)
Supplement: Supplementary file 1 [file S2059866121000261sup001.docx]

**SUPPLEMENTAL MATERIALS**

**Dataset References**

1. . N3C - https://ncats.nih.gov/n3c

2. Critical care registry: https://www.sccm.org/Research/Research/Discovery-Research-Network/VIRUS-COVID-19-Registry

3. Cancer and COVID registry: https://ccc19.org/

4. American Cancer Society: https://www.facs.org/quality-programs/covid19-registry

5. American Heart Association:  <https://www.heart.org/en/professional/quality-improvement/covid-19-cvd-registry>

**Survey Questions**

1. Does your organization have a dedicated data warehouse for research?
2. ( ) Yes
3. ( ) No

1.1. What is the Common Data Model (CDM)? (Check all that apply)

1. [ ] i2b2/ACT
2. [ ] PCORNet
3. [ ] TriNetX
4. [ ] OMOP
5. [ ] Other

1.2. What was the refresh frequency pre-COVID-19?

1. ( ) Daily
2. ( ) Weekly
3. ( ) Monthly
4. ( ) Other

1.3. How did this frequency change during the pandemic?

1. ( ) More Frequent
2. ( ) Less Frequent
3. ( ) Stayed the Same

1.4. Please describe the resources for querying and analyzing the data.

1. Are your researchers able to do rapid cohort identification of COVID-19 patients?
2. ( ) Yes
3. ( ) No
4. Has your organization developed a COVID-19 Data Mart/Registry and/or dashboard?
5. ( ) Yes
6. ( ) No

3.1 Please select what the COVID-19 Data Mart/Registry and/or dashboard is used for. (Check all that apply)

1. [ ] Predictive Model Development
2. [ ] Clinical Trials
3. [ ] Operations
4. [ ] Other
5. Have you seen an increase in the utilization of other informatics resources, such as REDCap, data warehouse, CTMS, management systems, etc. due to COVID-19?
6. ( ) Yes
7. ( ) Stayed the same
8. ( ) No, we experienced a decrease

4.1 Please describe the resource and how it was used during COVID-19.

1. Does your organization (Enterprise or CTSI) have a review committee(s) to manage the process for prioritization of access to EHR data?
2. ( ) Yes
3. ( ) No

5.1 Did this committee exist pre-pandemic?

1. ( ) Yes
2. ( ) No

5.1a How did forming this committee help with COVID-19 research?

5.1b Please describe the committee members' areas of expertise and roles.

1. In what capacity has your organization streamlined process for the following activities? (One choice per row)

| **One choice per column** | | | **COLUMN** | | | | |
| --- | --- | --- | --- | --- | --- | --- | --- |
|  |  |  | **A** | **B** | **C** | **D** | **E** |
|  |  |  | **The process existed prior to the pandemic and adapted that process to support continuation of COVID-19 research during the pandemic** | **Implemented a new process as a result of the pandemic** | **Implemented a new process as a result of the pandemic AND will continue to use this process once research operations resume to normal** | **Streamlined a new process, but was not effective** | **No process exists at your institution** |
| ROW | **A** | **Data Request** | ( ) | ( ) | ( ) | ( ) | ( ) |
|  | **B** | **Review/Approval** | ( ) | ( ) | ( ) | ( ) | ( ) |
|  | **C** | **Delivery** | ( ) | ( ) | ( ) | ( ) | ( ) |

6.1. How did creating this process help with COVID-19 research?

6.2 Please describe the existing/newly implemented process and its impact on the COVID-19 research.

1. Describe any innovations that have emerged at your site due to COVID-19 in regards to informatics.
